# Attention-Deficit Hyperactivity Disorder in Adults Using Methamphetamine: Does It Affect Comorbidity, Quality of Life, and Global Functioning? 

**Published:** 2018-04

**Authors:** Ronak Mihan, Zahra Shahrivar, Javad Mahmoudi-Gharaei, Alia Shakiba, Mostafa Hosseini

**Affiliations:** 1 Psychiatry Faculty, University of Social Welfare and Rehabilitation Sciences, Tehran, Iran.; 2 Department of Psychiatry, Tehran University of Medical Sciences, Tehran, Iran.; 3 Research Center for Cognitive and Behavioral Sciences, Tehran University of Medical Sciences, Tehran, Iran.; 4 Psychiatry and Psychology Research Center, Tehran University of Medical Sciences, Tehran, Iran.; 5 Department of Epidemiology and Biostatistics, School of Public Health, Tehran University of Medical Sciences, Tehran, Iran.

**Keywords:** *Adult*, *Attention Deficit Hyperactivity Disorder*, *Comorbidity*, *Function*, *Methamphetamine*, *Quality of Life*

## Abstract

**Objective:** Attention-deficit hyperactivity disorder (ADHD) is common in adulthood, and it is associated with different high- risk behaviors, particularly substance use. Evidence suggests a high prevalence of ADHD in adults who take methamphetamine (METH). This study aimed at comparing functional level, quality of life, and psychiatric comorbidities in METH users with and without adult ADHD (A-ADHD).

**Method** **:** In this cross-sectional study, 134 patients who had a history of METH use (at least once in lifetime) were selected from among inpatient and outpatient referrals to a psychiatric hospital. DIVA was performed for those who were positive on the Conners' Adult ADHD Rating Scales–Self-Report-Screening Version (CAARS-SR-SV). The Global Assessment of Functioning (GAF) and World Health Organization Quality of Life Scale-Brief (WHOQoL-BREF) were used to assess the participants’ level of functioning and quality of life, respectively. Psychiatric comorbidities including substance use disorders were evaluated using the Structured Clinical Interview for DSM-IV-Axis I (SCID-I).

**Results:** Among the METH users, 10.4% were diagnosed as having A-ADHD. A-ADHD was more prevalent among female METH users than males. The hyperactive-impulsive and combined types were more common than the inattentive type. Opiates and cannabis were the most commonly abused drugs by the 2 groups, while sedative-hypnotic use was significantly higher in the individuals with A-ADHD. Substance-induced mood disorder was the most prevalent comorbidity in the 2 groups and was higher in those with A-ADHD. quality of life and the GAF scores were significantly lower in those with A-ADHD and duration of METH use was higher Compared to the METH users without A-ADHD, (p>0.05).

**Conclusion: **This study provided some preliminary findings supporting the prevalence of Adult ADHD among METH users and its negative impacts on their global functioning and quality of life. To provide more effective intervention for METH users, detection and treatment of those with A-ADHD can be of clinical value.

Attention-deficit hyperactivity disorder (ADHD) is a common and disabling mental health problem in adults, with a reported prevalence of up to 5% in the general population (1). Approximately 75% of adults with ADHD suffer from other psychiatric comorbidities, such as learning disabilities, anxiety or mood disorders, sleep disorders, personality disorders, and substance use disorders (SUDs) (2). 

Adult ADHD (A-ADHD) is associated with a substantially higher risk of a lifetime history of nicotine or illicit drug use (3).

The higher rate of comorbid substance use with A-ADHD has been due to some shared genetic components (4), or shared neural substrates (5) both related to dopamine neurotransmission and behavioral profiles (i.e., impulsive behavior). On the other hand, the negative consequences of ADHD itself (poor performance, lower achievements, and deviant peer groups) have led to METH 

abuse (6, 7). Some studies, however, suggest that the higher risk for METH use among those with A- ADHD is mediated by other comorbid disorders (conduct or bipolar disorder) (8).

Psychostimulants are among the first-line medications of choice. However, there is an important controversy regarding the stimulant use for adult ADHD in substance-abusing populations. While some authors are concerned about the risk of psychostimulant medication abuse and dependence, methylphenidate has shown to be effective in decrease of substance use, recurrence, and craving (2). However, amphetamine-type stimulants including METH is a major concern, as it is the second most widely used substance after marijuana, according to a report from the United Nations Office on Drugs and Crime (9). METH is a potent and addictive stimulant used by over 400 000 Americans every year, and its serious neuropsychiatric and psychosocial consequences often lead to disability (10). In Iran, METH is called “shisheh” or crystal (literal translation of ‘Ice’) and is sold in powdered form and it it usually smoked. Data from the Iranian Drug Control Headquarters suggest a rapidly increasing METH use among young adults. In 2008, over 6% of substance abusers aged 12 years or older in Iran were current METH users, and use was most prevalent among young adults (11). 

 One important predictor of disability, which is commonly comorbid with METH use, is ADHD (12). Existing literature have mostly focused on METH use problems in adults with ADHD, and studies on A-ADHD in METH users in Iran are scarce. With regards to the high prevalence of METH use and ADHD in young adults, the prominent comorbidity of ADHD and substance use, and the negative consequences of both conditions, this study was conducted to assess the prevalence of ADHD in adults with METH use who referred to a psychiatric hospital. Moreover, psychiatric comorbidities, quality of life, and global functioning of the participants with and without ADHD were evaluated.

## Materials and Methods

*Participants and Procedure*

This cross-sectional study was conducted among all outpatients or admitted referrals to Roozbeh psychiatric hospital in Tehran from June 2015 to October 2016. The participants were 18 to 65-year-olds who reported a history of at least one-time METH use in their lifetime. They were not included if they had any cognitive disorders (eg, dementia) or intellectual disability. In case of acute intoxication or withdrawal of substances or delirium, the assessments were postponed to a later time when the participant was ready to cooperate with the study assessments. 

A board-certified psychiatrist evaluated the psychiatric diagnoses of all participants including substance use problems. Then, to screen ADHD symptoms, confirm the diagnosis, and check comorbidities, the patients were referred for further clinical evaluation using the Structured Clinical Interview for DSM-IV Axis I Disorders (SCID I). The participants filled the Conner’s Adult ADHD Rating Scale Self-report- Screening Version. For those who had the score of 55 or higher, the semi-structured Diagnostic Interview for Adult ADHD (DIVA) was performed by a trained resident of psychiatry to check if the criteria of A-ADHD were fulfilled. To ensure the accuracy of information, if any of the family members were available, they were asked to come to the hospital and answer the questions of the DIVA interview. The patients were asked to complete the World Health Organization Quality of Life Scale-Brief (WHOQOL BREF) to assess their quality of life. The Global Assessment of Functioning (GAF) was also used to rate the level of general functioning.

This study was approved by the ethical committee of Tehran University of Medical Sciences. The aims and process were introduced and informed consent was obtained from all participants. The study did not intervene on the medication or non-pharmacologic treatments being provided to the participants. The participants were allowed to quit the study whenever they wanted.

**Instruments**

*Conner’s Adult ADHD Rating Scale- Self-report: Screening Version (CAARS-SR-SV)*

 The CAARS-S: S provides a useful dimensional evaluation system for both research and clinical use (13). It is a 26-item questionnaire, and the items are rated on a Likert-type scale (0 = not at all, 3 = severe) according to the patients’ current functional status (14). Arabgol et al. validated the Persian version of the CAARS-S: S and found the Cronbach’s alpha coefficient of all the subscales to be higher than 0.8 (15).

*Structured Clinical Interview for DSM-IV Axis I Disorders (SCID I)*

The SCID-I is a widely used semi-structured diagnostic interview to assess the presence of mental disorders based on DSM-IV criteria for axis I disorders. It was first developed after the publication of DSM-III and was then updated according to DSM-IV and DSM-5. The interview takes approximately 1 hour to complete (16). Sharifi et al. conducted the Persian version of SCID-I in a multi-center study and supported its validity for clinical and especially research purposes (17).

*Global Assessment of Functioning (GAF)*

The GAF was first introduced in 1987 to rate Axis V of the DSM-IV and describes symptoms and functioning. GAF is a 100-point scale, with 10-point intervals. The most severely poor functioning patient is described with 1 to 10 and the healthiest with the score interval of 91 to 100(18).

*Diagnostic Interview for Adult ADHD (DIVA)*

DIVA is a semi-structured diagnostic interview to assess adult ADHD and provides a thorough evaluation based on the diagnostic criteria of DSM-IV-TR. The DIVA has 2 parts: the first part deals with the core symptoms using a list of concrete and realistic examples for each criterion and the second part deals with the functional impairment due to symptoms in 5 areas. DIVA has been reported to be a reliable diagnostic tool for clinical and research purposes (19). The Persian version of DIVA has been evaluated in a study in Iran (20). 

*World Health Organization Quality of Life Scale-Brief (WHOQOL BREF)*

The WHOQOL BREF is a 26-item questionnaire extracted from the 100-item World Health Organization Quality of Life Scale. It requires 10 minutes to administer and assesses 4 domains of quality of life. Each item is scored on a 5-point Likert Scale, from 1 (not at all, very dissatisfied, and very poor) to 5 (an extreme amount, very satisfied, and very good). The WHOQOL-BRIEF has been validated in the general population and also in various patient groups including smokers and alcoholics (21). Nedjat et al. conducted the Persian version of WHOQOL BREF Tehran and provided some preliminary evidence of reliability and validity (22).

*Statistical Analysis*

Descriptive statistics were expressed in mean and standard deviation or percentages as appropriate. Differences in characteristics between participants were assessed by t tests. Prevalence of AADHD, its subtypes, and other Axis I disorders were estimated for the total sample and for each gender separately; moreover, gender differences were tested by exact chi- square test. Regression analyses were used to investigate associations with the patterns of substance use. Also, Pearson correlation was applied to consider the association of quality of life and other variables. P-values were considered significant at the level of p < 0.05. Data were analyzed using IBM SPSS Statistics 21.0 software.

## Results

A total of 134 patients (86.4% males, 50.6% single), with the mean age of 34.89(SD = 8.41) years, were enrolled in the study. Among them, 10.4% had been diagnosed as A-ADHD. Hyperactive-impulsive, combined, and inattentive presentation were found in 35.7%, 35.7%, and 28.5% of the participants, respectively. No significant differences were found between the groups in age, gender, education level, occupation, and marital status although the rate of A-ADHD in females was higher than in males (Table 1). Among the participants with A-ADHD, 57.1% had one or more medical condition, while this rate in those without A-ADHD was 46.7%.

Table 2 demonstrates substance use features in the participants. The age of first use and abuse/dependence was determined using the SCID-I items. Age of first amphetamine use and rate of lifetime drug dependency or abuse were higher among those with A-ADHD than those without it; however, the difference was not significant. The prevalence of lifetime and current concurrent other substance use was equal among the both groups, except for sedative-hypnotics, which was higher among the group with A-ADHD (P = 0.01). The most commonly used substances in the both groups were opiates, cannabis, and sedative-hypnotics. Those with A-ADHD were more dependent and used significantly more sedative-hypnotic medications compared to the group without A-ADHD (P value: 0.01). Mean duration of METH use in the participants with and without A-ADHD was 46.92 and 39.78 months, respectively, and correlated with their first age of any substance use.

Of those with A-ADHD, 50% had more than 3 comorbid disorders, while half of the other group had only 2 concurrent disorders. Rate of comorbidity in all participants was 0 in 1.5%, 1 in 23.1%, 2 in 35.8%, 3 in 27.6%, 4 in 9.7%, and 5 in 2.2%. The most common disorders were substance- induced mood disorders, mood disorders, psychotic disorders, and substance- induced psychotic disorders. Substance- induced mood disorder was more common in participants with A-ADHD. Almost all (98.4 %, N = 131) the participants met the criteria for at least one other Axis I disorder (100% of those with A-ADHD; 98.34% of those without A-ADHD). Table 3 demonstrates the rate of current and lifetime disorders in the 2 groups.

In global functioning, those with A-ADHD obtained lower scores on the GAF scale than those without A-ADHD although the difference was not significant. Moreover, 50% of all participants had the GAF score of lower than 20 to 30.

Finally, we did not find any significant difference between those with and without A-ADHD in WHOQOL scores (Table 4). WHOQOL subscale scores were correlated with some features of substance use. Physical quality of life was negatively correlated with age (p = 0.047) and age of first use of amphetamine (p = 0.034). It was lower among the participants who concurrently used sedative-hypnotics (p = 0.022), and higher in those with cocaine use (p = 0.009). Psychic quality of life was negatively correlated with the severity of ADHD (p = 0.027) and was lower in those who used sedative-hypnotics (0.002). Environmental quality of life was higher in males than in females and in those who were single or married than those who were divorced.

**Table 1:** Comparison of Demographic Characteristics Between the Participants with and Without A-ADHD

**Participants**	**With A-ADHD (N=14)**	**Without A-ADHD (N=120)**	**P value**
	**Frequency (%)**	**Frequency (%)**	
Gender Male female	10(71.4) 4(28.6)	106(88.3) 14(11.7)	0.096
Education Under diploma Diploma Higher than diploma	12(85.7) 0 2(14.3)	103(58.8) 9(7.5) 8(6.7)	0.363
Job Unemployment Self-employment Student Employee	8(57.1) 5(35.7) 1(7.1) 0	88(73.3) 29(24.2) 1(0.8) 2(1.7)	0.237
Marital Status Married Divorced Single Separated Widow	2(14.3) 5(50) 4(28.6) 1(7.1) 0	24(20) 23(19.2) 64(53.3) 8(6.7) 1(0.8)	0.116

**Table 2:** Substances Use Features in the Participants with and Without A-ADHD

**Substance Use Features**	**With A-ADHD (Mean ** **+** ** SD)**	**Without A-ADHD (Mean ** **+** ** SD)**	**P**
age of first use of any substance (y)	12 (22.64+9.05)	12 (22.54+6.83)	0.946
age of first use of METH (y)	17 (28.42+8.49)	13 (29.12+8.66)	0.776
Age of substance abuse or dependency (y)	12 (22.61+6.23)	13 (22.3+4.66)	
duration of METH use (month)	46.92± 35.57	42.98± 39.78	0.556
Substance	Frequency (%)	Frequency (%)	P value
Cannabis	8 (57.1)	67(55.8)	0.972
Cocaine	0	12(10)	0.364
Hallucinogen	1(7.1)	11(9.2)	0.9
Inhalant	0	0	
Opiates	12(85.7)	105(87.5)	0.692
PCP	0	0	
Sedative- hypnotic	7(50)	21(17.5)	0.01
Others	0	1(0.8)	0.931
Alcohol	7(50)	68(56.7)	0.635

**Table 3:** Current and Lifetime Comorbid Disorders in Participants with and Without A-ADHD

**Comorbid Disorder**	**Current Disorder**		**Lifetime Disorder**	
	**Without A-ADHD ** **(%)**	**Without A-ADHD ** **(%)**	**Without A-ADHD ** **(%)**	**With A-ADHD ** **(%)**
Any bipolar disorder	35.6	69.12	35.6	58.9
Any depressive disorder	28.6	8.4	14.2	1.6
Any psychotic disorder	14.2	19.9	14.2	15.8
Substance induced mood disorder	42.9	24.2	21.4	18.3
Substance induced psychotic disorder	14.2	15.8	21.3	9.2
Substance disorder	7.1	3.3	42.8	30
Alcohol dependency	0	0	0	1.7
Dependency cannabis	0	0	7.1	1.7
Amphetamine dependency	7.1	0.8	28.6	8.3
opiates dependency	0	2.5	7.1	17.5
Amphetamine abuse	0	0	0	0.8
No disorder	0	8.3	0	5

**Table 4:** Quality of Life in Participants with and Without A-ADHD Based on the WHOQOL BREF

**Quality of life**	**With A-ADHD (Mean ** **+** ** SD)**	**Without A-ADHD (Mean ** **+** ** SD)**	**P**
Physical	56.88±23.33	63.51±14.7	0.139
Psychic	47.02±19.84	53.36±17.92	0.217
Social	37.5±24.83	43.12±16.44	0.256
Environmental	57.36±16.1	58.54 ± 13.69	0.766

## Discussion

The present study was conducted to evaluate the prevalence of adult ADHD (A-ADHD) in methamphetamine (METH) users and compare global functioning, quality of life, and comorbidities in those with and without A-ADHD.

We found the prevalence rate of A-ADHD to be as high as 10.4% among METH users. This rate was up to 3 times higher than in Iranian general population; Arabgol et al. showed that 3.7% of Iranian university students were diagnosed as A-ADHD (15). However, the rate of A-ADHD in our participants was lower than the prevalence reported by Obermeit et al. (21) and Dakwar et al. (23). They found a prevalence of 20.8% among chronic methamphetamine users (24) and 25% among adults seeking treatment for cocaine use, respectively. It is noteworthy to mention that to enroll the participants, we recruited the individuals with at least one-time METH use, which is not the criteria for abuse or dependence. This can explain the lower rate of A-ADHD in the recent study compared to other researches. Among the individuals with A-ADHD, we found nearly equal prevalence of ADHD subtypes. Some research reported the combined type as the most common form of ADHD in adults (25, 26), while others found the inattention predominant subtype or hyperactive-impulsive subtype as the most frequent (27). It has been reported that the higher rate of substance use problems are seen in patients with combined type (25, 28).

The rate of A-ADHD among females with METH use was insignificantly higher than in males in the recent study. Disney et al. reported the higher odds ratio for nicotine and cannabis dependence in ADHD-affected girls than in ADHD-affected boys (29). 

We found higher rates of divorce and separation among the participants with A- ADHD, a fact consistent with preceding findings (15, 30). This supports the negative effects of A- ADHD on marriage and intimate relationships, leading to familial instability. 

Age of first substance use was the same in our participants, both with and without A-ADHD, while age of diagnosis of substance dependence correlated with the presence of A-ADHD and higher percentage of the individuals with A-ADHD (92.8% vs. 75.9%) met the criteria for substance dependence. Some previous studies reported that the first age of substance use was not different in groups with and without A-ADHD, however, those with A-ADHD started regular substance use at a younger age and had higher probability of substance dependence and shorter intervals between the age of first use and the age of diagnosis of abuse or dependence (8, 12). Nonetheless, 2 studies among Iranian population reported lower age of first substance use and dependence in participants with A-ADHD (12, 31). Some striking differences between study populations including age or recruitment source could contribute to the different findings. 

Beiderman et al. reported that cannabis was the most common substance in individuals with ADHD (8). There are other studies that found no preference of substances in those with ADHD (32, 33). Kousha et al. reported the sequence of cannabis, opium, and heroin in a group of Iranian adolescents with SUD and ADHD (34). In our study, opiates were the most common substance used by both groups followed by cannabis and sedative-hypnotics. Sedative-hypnotic drugs use among those with A-ADHD was higher than the control group. It can be suggested that individuals with A-ADHD have higher level of anxiety and that sedatives use represents an attempt to self-medicate the symptoms (12). Besides, dependency to cannabis and amphetamine was higher and dependency to opium was lower in our participants with A-ADHD compared to the other group. 

The higher rate of concurrent comorbid disorders among those who suffered from A-ADHD was consistent with previous studies, showing higher rates of concurrent psychopathology (32, 33). Substance- induced mood disorder was the reason for admission and more common in the participants with A-ADHD. This is in line with the fact that they were hospitalized and suffered from substance- induced psychiatric disorders much more than the METH users without ADHD (5, 10). This finding confirms the need to screen the individuals with METH use for probable comorbid ADHD and the challenge it imposes on therapeutic interventions. Individuals with A-ADHD were diagnosed as having depressive disorders more than those without A-ADHD, while this was reverse in bipolar and psychotic disorders. 

Previous studies reported lower GAF scores in patients with A-ADHD than those without it (12). In this study,

lower scores in GAF and each of 4 domains of quality of life were found in those with A-ADHD. Sedative-hypnotics use decreased psychic and physical quality of life; with increasing first age of METH and sedative-hypnotics use, physical quality of life reduced. Unexpectedly, cocaine use was associated with improvement in physical quality of life, but cocaine use in those participants was transient and brief (no history of abuse or dependency was detected). However, to explain these findings, additional studies are needed. Higher percentage of persons with a history of physical disorders in those with A -ADHD in comparison to non A-ADHD group confirms that common health problems and diseases in adults with ADHD can also affect their quality of life. Our findings showed that environmental quality of life in single and married individuals was higher than in divorced or separated participants and in males was superior to females. Difference in all subscales of Quality of Life Enjoyment and Satisfaction Questionnaire (Q-LES-Q) in patients with ADHD has been already shown and represents low quality of life in participants with A-ADHD (35).

## Limitation

This was one of the limited studies on A-ADHD in METH users, while most studies have been conducted on the METH use in adults with ADHD. There are possible reasons for different findings in our study including different types of instruments used for A-ADHD evaluation, sample size, and methods of sample recruitment in different studies. We used both self-report questionnaire and structured interview to check the psychiatric diagnoses, while in some research the diagnosis was made by screening measures. 

However, the findings should be considered in light of some limitations. First, the participants were selected from a tertiary hospital whose referrals come from different regions of the city and even the country with diverse psychosocial background and ethnicities. They usually suffered from more severe psychiatric disorders and higher rate of comorbidities, and this might lead to selection bias and might also limit the generalizability of our findings. Second, self-report measurements of quality of life may be subject to bias. Third, the statistical analysis might have been affected by the small number of individuals with A-ADHD among the participants, which led to an unbalanced distribution of sample size. Finally, the predominance of male participants in the study may hinder the generalizability of the findings.

## Conclusion

This study supports the fact that ADHD in adults can affect their tendency to use illegal drugs and substances. A-ADHD also appears to play an important role in METH-use associated disability including declines in quality of life and global function level. Moreover, it increases the rate of concomitant psychiatric disorders. Targeted ADHD screening and treatment can help improve real world outcomes for individuals with METH use disorders.
